# Data on steam inhalation in combating CoVid-19

**DOI:** 10.6026/97320630018825

**Published:** 2022-09-30

**Authors:** Barun Dev Kumar, Santosh Kumar Verma, Akhilanand Chaurasia, Deepyanti Dubey

**Affiliations:** 1 Dental Institute, RIMS, Ranchi, India

**Keywords:** COVID-19 infection, SARS-CoV-2, steam inhalation, CO-RADS

## Abstract

Steam inhalations are often used to control viral infections of the respiratory tract such as common cold. The use of steam inhalation in combating SAR-CoV-2 infection has been also tried. Therefore, it is of interest to evaluate the various data available on the effect of steam inhalation on COVID-19 infection in a systematic manner. The guidelines of the Preferred Reporting Items for Systematic Reviews and Meta-Analyses (PRISMA) were used. We registered the protocol in PROSPERO, the International prospective register of systematic reviews. A search method to identify relevant studies using PICO questions was prepared. A total of 52 articles were screened for their relevance to the topic. Three articles were found to have insufficient data and ten articles could not pass our inclusion criteria. Total 3 articles could make the final list of articles based on inclusion and exclusion criteria. Steam inhalation helps in symptomatic relief of COVID symptoms. But there is not much data available to reach a conclusion of its role in the treatment and prevention of COVID.

## Background:

Inhalation of different herbal additives in boiling water like eucalyptus, Tulsi, Bay leaf, Camphor, Cinnamon, and Neem have beneficial effects in the treatment of respiratory problems like common cold [1], bronchitis [2], asthma, and chronic obstructive pulmonary disease (COPD) [3]. The antimicrobial properties of these herbs help to fight infection and give a sense of well-being. Today, the world is facing one of the most deadly pandemics in the history of mankind which is caused by novel virus called severe acute respiratory coronavirus 2 (SARS-CoV-2) [4]. Many drugs or treatment modalities have been tried to treat SARS-CoV-2 infection but till now a definite treatment protocol for curing the COVID-19 infections has not been established [5]. High temperature of steam can result in irreversible denaturation of proteins of SARS CoV and loss of its infectivity [6,7]. Inhalation of hot air also improves breathing by enhancing the mucociliary clearance [8]. It reduces pulmonary congestion and improves respiratory function along by increasing tidal volume, vital capacity, ventilation, and forced expiratory volume of the lungs [9]. Steam inhalation also improves cardiovascular function, blood pressure, increasing cardiac output, plasma volume, and peripheral blood flow [10-11]. An in vitro study has demonstrated that direct application of heat at 45°C for 20 minutes to the upper airways, activates immune cells, releasing heat shock proteins and suppressing rhinovirus multiplication by more than 90% [13]. Therefore, it is of interest to document known data Steam inhalation in combating CoVid-19.

## Materials and Method:

The guidelines of the Preferred Reporting Items for Systematic Reviews and Meta-Analyses (PRISMA) were followed. We registered the protocol in PROSPERO, the International prospective register of systematic reviews. A search method to identify relevant studies using PICO questions was prepared.

## Population:

COVID-19 patients of any age, with any co-morbidity, and any level of severity.

## Intervention:

Inhalation of steam or water vapor or thermal inactivation delivered through any method, with or without admixed medicine.

## Control:

Controls received placebo or no intervention.

## Outcomes:

Studies that included any of the following outcomes were included

[1] Improvement in signs and symptoms (e.g. increased oxygenation, decrease in respiratory distress, decrease in symptom scores, reduced symptomatic duration)

[2] Microbiologic resolution (e.g. decreased viral load/shedding, negative cultures)

[3] Improvement in airway mechanics (e.g. decreased nasal resistance, increased lung volumes)

[4] Safety of steam inhalation (e.g. scald burn, nasal irritation, etc.)

## Review question:

Is steam inhalation effective in the treatment of COVID 19 patients? What is the efficacy and safety of steam inhalation for the treatment and prevention of COVID-19?

## Literature search methods:

Search strategy:

Articles were searched (up to May 2021) of Pubmed, OVID, Embase, Science Direct, Google Scholar, and Web of Knowledge for studies reporting usage of steam inhalation in the treatment of COVID-19 patients. In addition, the reference lists of relevant articles and review articles were also searched to collect more relevant studies. Only articles published in English were considered (Figure 1 - see PDF). Two search words were combined using the Boolean operator 'and'. The first theme, steam inhalation, combined (MeSH) thermal inhalation, water vapor inhalation, mist inhalation. The second term, covid -19, combined exploded versions of MeSH terms coronavirus or SARS-CoV-2 (Table 1 - see PDF).

## Selection criteria:

Two investigators (S.V. and B.D.K.) searched and checked for the eligibility of the article independently by and disagreements were resolved by another investigator (D.) All the duplicate articles were removed, and then abstracts and full contents of articles were properly screened for eligibility using inclusion and exclusion criteria.

## Inclusion and exclusion criteria:

Articles were included in the meta-analysis if: (1) the authors presented an original, peer-reviewed study; (2) the study was a case-control study or randomized control trials, a prospective study. (3) Moreover, studies were included when COVID 19 patients had any other systemic disease or any medications were also assessed. Excluded article: (1) articles like meeting reports or review articles, newsletters, conference abstracts, protocols, press reports, opinion articles, or in-vitro studies were excluded. (2) Studies were excluded when sufficient data were not available.

## Results

A PRISMA flow chart (Figure 1 - see PDF) represents the result of searching for relevant articles in various search engines. A total of 52 articles were screened for their relevance to the topic. Three articles were found to have insufficient data and ten articles could not pass our inclusion criteria. Total 3(see PDF) articles could make the final list of articles based on inclusion and exclusion criteria. All the articles and their details, study design parameters, outcome, and result are represented in Table 2(see PDF).

## Included articles:

La Marca et al. [13] (2021) in a single-centered open-label interventional study on 10 asymptomatic or pauci-symptomatic healthcare professionals with rhino-pharyngeal (RN) swab showed positive for SARS-CoV-2 infection. Patients were advised to take humidified steam for at least 20 min (5 cycles of 4 min) within 1 h for at least 4 consecutive days. The reduction of viral shedding was measured by real-time PCR on rhino-pharyngeal (RN) swab sampling 24 h after each cycle of treatment. 6 patients with symptoms reported clinical improvement at the end of protocol: 3 patients reported to be cleared of all symptoms; 2 declared persisting anosmia and ageusia; one reported mild muscle pain and nasal congestion. All seven patients achieved both outcomes, testing negative after the first day of steam inhalation (4 consecutive RN swabs).

Swain et al. [16] (2021) conducted a study on 52 asymptomatic and 44 symptomatic COVID-19 patients. 52 asymptomatic patients were advised for inhaling steam thrice daily for two weeks, the duration of each inhalation was 5 minutes. All these 52 patients became negative for COVID-19 infection during the second weeks with (RT-PCR) test. Out of 44 symptomatic COVID-19 health care professionals 36 had mild symptoms and 8 had moderate symptoms. These patients were advised to take steam inhalation every three hours and each inhalation for 5 minutes along with COVID-19 treatment in the COVID hospital. All the patients were visited for follow-up at 1st week, 2nd week, one month, and two months. All the mild symptomatic COVID-19 patients became symptom-free in 5 days with steam inhalation whereas in moderate symptomatic patients (except one patient), all of them became symptom-free after 7 days. One moderate symptomatic patient has developed pneumonia followed by orotracheal intubation and this patient was admitted to the COVID intensive care unit (ICU) and discharged for home quarantine. The CT thorax is high sensitivity and specificity for an immediate result. CT scan of the lungs shows infiltrates, ground-glass opacities, and segmental consolidation. In this study, the asymptomatic patients were showing the score of 0 to 5 in CT thorax before steam inhalation and became 0 after steam inhalation.

Mody [15] (2021) conducted a study on 60 COVID positive patients. All 60 patients received all therapeutic measures as part of covid-19 treatment protocol but 30 patients also received Sodium bicarbonate inhalation. 50 ml of 8.4% Sodium Bicarbonate vapor was delivered through a naso-oral delivery apparatus attached to an electrically operated steam generating apparatus. The administration was for 5 minutes per session. This was done at a frequency of twice daily 8 hours apart, for 5 days. Clinical parameters and were recorded on Day 1 and Day 5. Results showed a highly significant improvement in the clinical symptomatic picture of covid-19 affected patients when they were treated with inhalations of steam impregnated with 8.4% sodium bicarbonate. There was also a highly significant reduction in the CRP values of patients treated with steam inhalations impregnated with 8.4% sodium bicarbonate. Whilst there was a trend showing improvements in numerical values of other blood markers (ESR, LDH, IL6, D-dimer, and Ferritin).

## Discussion:

The COVID-19 infection is a highly contagious disease that spread rapidly from human to human via droplets; source of infection is either infected patients or asymptomatic carriers [16]. The transmission of the SARS-CoV-2 usually occurs through the upper respiratory tract such as the nose, nasopharynx, oral cavity, pharynx, and larynx with high levels of viral shedding [17]. The direct application of steam to the upper airways, routinely or at the first signs of infection, may further serve to inhibit or deactivate virions in the place where they first lodge. It may reduce viral shedding and reduce the transmission of the illness. This may break the chain of infection. Approximately 80% of patients who contract COVID- 19 develop only mild flu-like symptoms and 79% of the reported cases got infected from other symptomatic or asymptomatic patients of COVID-19 infections [18]. Steam inhalation may be useful especially in high risk subgroup with comorbid conditions and above 60 years of age. This has been demonstrated in vitro with temperatures of 45°C for 20 minutes activating immune cells, releasing HSPs, and suppressing rhinovirus multiplication by more than 90% [19]. The inhalation of steam with added essential oils with anti-viral, decongestant, anxiolytic, and other properties, may further assist in facilitating mucociliary clearance and reducing viral load as well as providing physical and psychological relief [20]. The traditional Indian medicinal system of curing common cold has shown a beneficial effect of herbal steam inhalation for years. Ministry of AYUSH under Govt of India has given the recommendation for the usage of herbs with medicinal benefits like Pudina (Mint) leaves, Ajwain (Caraway seeds), Lavang (Clove) in steam for immunity boosting and strengthening the respiratory system during home isolation [21]. Patients who have reported respiratory problems can get relieved of their symptoms by taking herbal steam inhalation. A study where herbal steam inhalation was used as a complementary therapy in addition to antibiotics, vitamins, and minerals reported early recovery in patients with the viral common cold [22-23]. There are currently no clinical protocols for using heat in the treatment of COVID-19, yet it is found beneficial in covid symptoms like, cough, cold, and flu . A study has shown to reduce coronavirus infectivity by at least 4 log10 when the temperatures in a sauna exceed 60°C for 30 min, 65°C for 15 min or 80°C for 1 min [24]. While the temperature, humidity and time required to specifically deactivating SAR-CoV-2 in vivo are yet to be determined.

## Inhalation process, recommendation:

We did not find any recommendations on the use of steam inhalation from the World Health Organization (WHO) and Centers for Disease Control and Prevention (CDC). The protocol for steam inhalation in several studies is almost the same. Hot boiling water is poured carefully into a bowl. Then the patient is advised to drape the towel over the back of the head and slowly bend toward the hot water until you are about 8 to 12 inches away from the water. Steam is inhaled slowly and deeply through the nose for at least two to five minutes. A timer is used to keep the proper frequency and duration of inhalation. Each session should not be longer than 10 to 15 minutes. The session can be repeated two or three times per day [8]. Any person who has returned home or in contact with other people is advised to inhale steam. For patients with COVID-19 infection, the frequency of steam inhalation therapy is modified based on the severity and symptoms of the infection. All these mild to moderate symptomatic patients were advised to take steam inhalation every three hours and each inhalation for 5 minutes along with COVID-19 treatment in the COVID hospital. For severe patients, the study protocol consisted of humidified steam through inhalation for at least 20 min (4 cycles of 5 min or 5 cycles of 4 min) within 1 h, with a temperature maintained between 55 and 65°C in the first 4/5 min after initiation of water boiling.

## Safety concerns of thermal inhalation:

A search of databases did not find any trial or research on burn injury due to steam inhalation in patients during COVID -19 pandemic. We found some reported cases of burns due to boiling water from hospitals. There are several studies reporting the thermal inhalation causes scald burn injury. A hospital in UK revealed an increase in cases of scald burn related to steam inhalation during the COVID-19 pandemic [25]. Within the first month of the pandemic, the hospital has admitted 6 pediatric cases of scald burns from steam inhalation, as opposed to their yearly average of only 2 cases. Vigorous inhalation causes injury to the respiratory tract, alveolar epithelium which results in increased pulmonary capillary permeability, pulmonary oedema, and interfering abnormalities in gas exchange [26]. Despite the considerable risk of paediatric scalds with steam inhalation, as highlighted in previous articles [27 -29]. This practice continues to be advocated in primary care.

## Conclusions:

There is insufficient evidence to support the use of steam inhalation for the prevention of COVID-19. Steam inhalation therapy can have severe adverse side effects, such as burn injuries due to overturning the bowl of steaming water. Accidental burns can also be prevented by adopting some precautions like use of commercially available steamer. More randomized control trials evaluating stream inhalation efficacy in preventing COVID-19 are needed.
